# Clinical, biochemical, and genetic characterization of acute hepatic porphyrias in a cohort of Argentine patients

**DOI:** 10.1002/mgg3.1059

**Published:** 2021-03-25

**Authors:** María del Carmen Martinez, Gabriela Nora Cerbino, Bárbara Xoana Granata, Alcira Batlle, Victoria Estela Parera, María Victoria Rossetti

**Affiliations:** ^1^ Centro de Investigaciones sobre Porfirinas y Porfirias (CIPYP) Hospital de Clínicas José de San Martín, CONICET‐UBA Buenos Aires Argentina; ^2^ Departamento de Química Biológica Facultad de Ciencias Exactas y Naturales – Universidad de Buenos Aires (UBA) Buenos Aires Argentina

**Keywords:** acute hepatic porphyrias, acute intermittent porphyria, heme metabolism, oxidative stress, variegate porphyria

## Abstract

**Background:**

Acute Hepatic Porphyrias (AHPs) are characterized by an acute neuroabdominal syndrome including both neuropsychiatric symptoms and neurodegenerative changes. Two main hypotheses explain the pathogenesis of nervous system dysfunction: (a) the ROS generation by autooxidation of 5‐aminolevulinic acid accumulated in liver and brain; (b) liver heme deficiency and in neural tissues that generate an oxidative status, a component of the neurodegenerative process.

**Methods:**

We review results obtained from Acute Intermittent Porphyria (AIP) and Variegate Porphyria (VP) families studied at clinical, biochemical, and molecular level at the CIPYP in Argentina. The relationship between the porphyric attack and oxidative stress was also evaluated in AHP patients and controls, to identify a marker of neurological dysfunction.

**Results:**

We studied 116 AIP families and 30 VP families, 609 and 132 individuals, respectively. Genotype/phenotype relation was studied. Oxidative stress parameters and plasma homocysteine levels were measured in 20 healthy volunteers, 22 AIP and 12 VP individuals.

**Conclusion:**

No significant difference in oxidative stress parameters and homocysteine levels between the analyzed groups were found.

## Graphical abstract

In this paper we review same results obtained from the AIP and VP families studied at biochemical and molecular level at the CIPYP in Argentina.Taking into account the relationship between the porphyric attack and oxidative stress, we also decided to study oxidative stress parameters and homocysteine levels in AHP patients and controls, to identify a marker of neurological dysfunction.

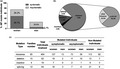

## 1 BACKGROUND

The porphyrias are a group of genetic metabolic disorders caused by the partial deficiency of one of the enzymes involved in the heme biosynthetic pathway. These disorders can be classified on the basis of their clinical manifestations into cutaneous, acute, and mixed porphyrias (Battle, 1997; Puy, Gouya, & Deybach, 2010).

Particularly, individuals with acute hepatic porphyrias (AHPs), where liver is the major site of expression of the enzymatic defect, suffer from neurovisceral attacks (Albers & Fink, 2004).

From the four AHPs, Acute Intermittent Porphyria (AIP) (OMIM: 176000) and Variegate Porphyria (VP) (OMIM: 176200) are the most common type of acute porphyria in our country.

AIP is an autosomal dominant disorder caused by a deficient activity of hydroxymethylbilane synthase (*HMBS*) (EC 4.3.1.8; OMIM: 609806), also referred to as porphobilinogen deaminase, producing a markedly increase in the urinary excretion of 5‐aminolevulinic acid (ALA) and porphobilinogen (PBG). The symptoms may frequently generally appear at any time after puberty and are characterized by intermittent attacks of abdominal pain, constipation, vomiting, hypertension, tachycardia, fever, and various peripheral and central nervous system manifestations (Albers & Fink, 2004; Puy et al., 2010).

VP is an autosomal dominant disorder associated to a deficiency of the penultimate enzyme of the heme biosynthetic pathway the Protoporphyrinogen oxidase (*PPOX*; EC 1.1.3.4; OMIM: 600923). Patients with VP may present a broad spectrum of clinical manifestations characterized by cutaneous photosensitivity and neurological symptoms which can occur separately or together in affected individuals. Cutaneous photosensitivity is characterized by skin fragility, erosions, blisters, millia, and pigmentary changes in sun exposed areas. Neurological symptoms are similar to those of AIP.

Acute attacks in latent individuals can be induced by a variety of environmental factors including many common medications, nutritional factors, restricted carbohydrate, and calories intake, hormones (Albers & Fink, 2004). A common mechanism of these triggering agents is to increase the hepatic heme demand as a consequence of the induction of their metabolizing enzymes, CYP450, which have heme as a prosthetic group. The hepatic heme pool is reduced and consequently the first and limiting enzyme of the pathway, 5‐aminolevulinic synthase (*ALAS1*; OMIM: 125290), is induced, so the synthesis of its product ALA is increased. In individuals with a partial deficiency in one of the enzymes of heme biosynthesis, the deficient enzyme becomes limiting and precursors (ALA and PBG) accumulate.

The biochemical events in AHPs are characterized by a massive accumulation and excretion of ALA and PBG during the neurovisceral attack. Even though ALA is neurotoxic, the exact mechanism by which this precursor carries out their action is not yet known and the pathogenesis of nervous system dysfunction remains unclear (Dwyer, Stone, Zhu, Perry, & Smith, 2006). There is a hypothesis which states that ALA can oxidase itself generating Reactive Oxygen Species (ROS) which increase the oxidative stress in the cell, thus explaining the porphyric attack (Onuki et al., 2004).

Another hypothesis is based on heme deficiency in liver and neural tissues impairing critical processes depending on hemoproteins (Dwyer et al., 2006). The heme deficiency can be a crucial component of the neurodegenerative process (Atamna, Killilea, Killilea, & Ames, 2001). Furthermore, there are alterations of heme biosynthetic pathway associated with Alzheimer's disease, where the oxidative damage is present since early stages with neurological dysfunction (Atamna & Frey, 2004). This is due to an oxidative stress as a consequence of an elevation of homocysteine levels in plasma which mobilizes iron storage from ferritin (Dwyer, Raina, Perry, & Smith, 2004).

In this paper, we review same results obtained from the AIP and VP families studied at biochemical and molecular level at the CIPYP in Argentina (Cerbino et al., 2015; Rossetti, Granata, Giudice, Parera, & Batlle, 2008). Taking into account the relationship between the porphyric attack and oxidative stress, we also decided to study oxidative stress parameters and homocysteine levels in AHP patients and controls, to identify a marker of neurological dysfunction.

## 2 MATERIALS AND METHODS

Informed consent was obtained from all patients following the standards of UNESCO Declarations‐DDHH Genome and Genetic Data (http://www.unesco.org/shs/ethics). Declaration of Helsinki was taken into consideration, and the study was approved by the Institutional Research Ethics Committee (CIPYP, CONICET – UBA).

From March 1994 to December 2016, 153 unrelated families, mostly from Argentina, were studied at biochemical and molecular level. All patients had current symptoms of AIP or VP and the diagnosis was made on the basis of their clinical history of at least one acute attack or, in VP patients, typical cutaneous lesions, associated with increased excretion of ALA, PBG, and total porphyrins in urine and plasma in AIP and also in feces for VP (Méndez et al., 2012). The final diagnosis of the patients was established by genetic studies. Unrelatedness was determined by family inquiries.

Genetic studies were done according to the methodologies described in Cerbino et al., 2015 and Rossetti et al., 2008.

The Human Gene Mutation Database (http://www.hgmd.cf.ac.uk/) was used for information about reported mutations in the *HMBS* and *PPOX* gen.

### 2.1 Oxidative stress and neurological damage parameters

Plasma was obtained from anticoagulated blood samples by centrifugation at 1500 *g* for 10 min. There were three groups included in the study: AIP individuals (*N* = 22), VP (*N* = 12), and healthy volunteers (*N* = 20).

The determination of oxidative state and antioxidant defenses where performed according to the following methods: the total oxidant state, Erel (2005); malondialdehyde levels (MDA), Grotto et al. (2007); oxidative damage to proteins, Reznick and Packer (1993); reduced glutathione (GSH) levels, Rossi, Cardaioli, Scaloni, Amiconi, and Simplicio (1995); the total antioxidant capacity, Erel (2004); Catalase activity, Chance and Maehly (1965). The determination of plasma homocysteine levels was performed according to the method of Krijt, Vackova, and Kožich (2001). Protein content was measured by Lowry, Rosebrough, Farr, and Randall (1951).

The experiments were performed in duplicate and results were expressed as the mean ± standard deviation. Statistical analysis was performed using one‐way ANOVA to determine the differences between groups. It was considered that the difference between the patient and the control groups was significant at *p* < .05.

## 3 RESULTS

### 3.1 Study of AIP families

**FIGURE 1:**
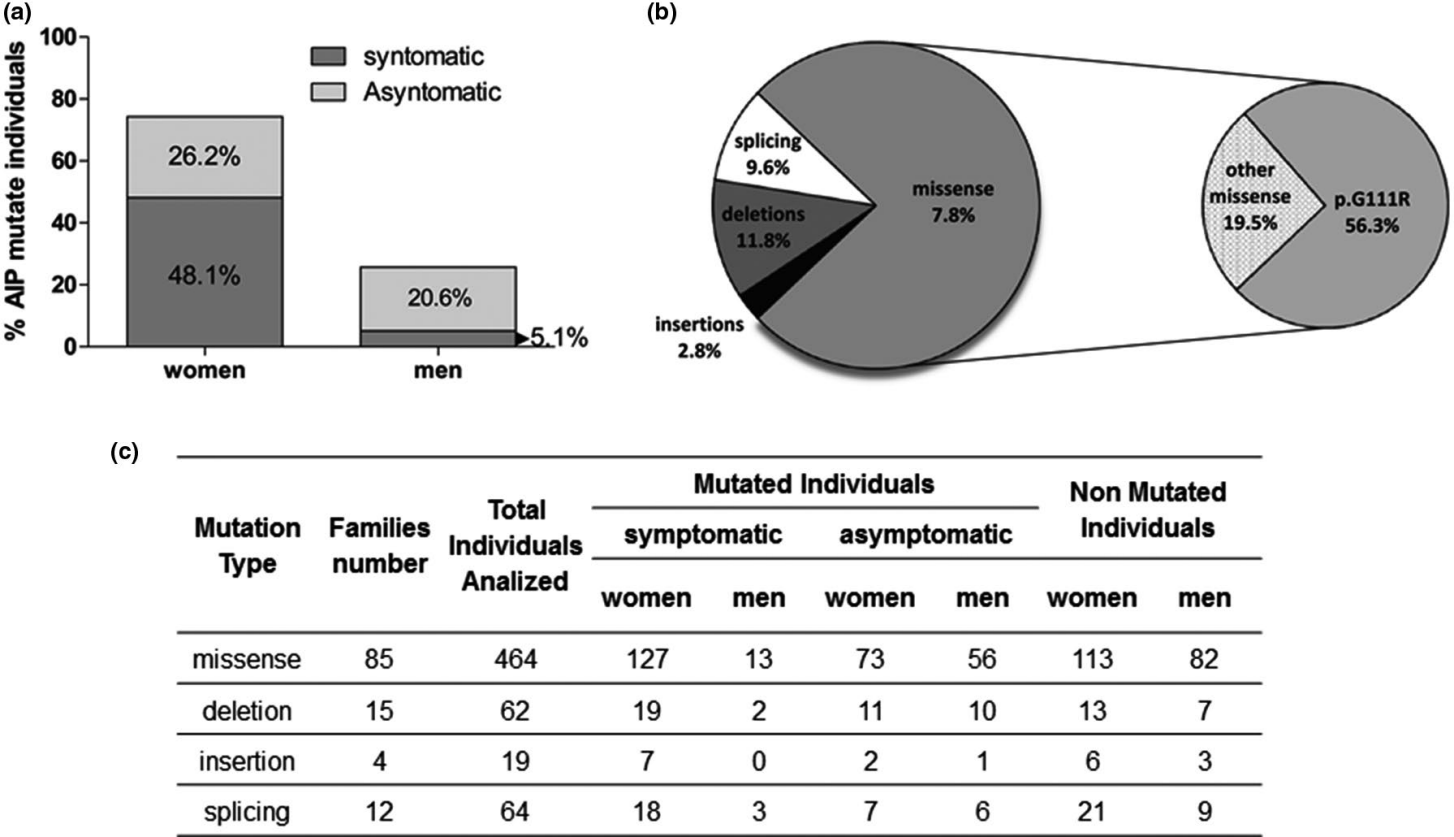
(a) Percentage distribution of AIP‐mutated individuals by sex and according to clinical and biochemical manifestation. (b) Percentage distribution of the four types of AIP mutations found in Argentina. The subgraph shows the distribution of p.G111R (NM_000190.4 (*HMBS*): c.331G>A (p.Gly111Arg)) mutation among the total missense mutations. (c) Distribution of individuals carrying an AIP mutation according to symptomatology, sex, and the type of genetic variant

During the last 30 years, 116 families were biochemically and genetically diagnosed as AIP at the CIPYP. We analyzed 609 individuals (417 women and 192 men).The results from this analysis were shown in Figure 1a,b.

Of the total missense mutations, p.G111R (NM_000190.4 (*HMBS*): c.331G>A (p.Gly111Arg)) corresponds to 72.3%, the most prevalent in Argentina (56.3%), for which we had described a founder effect (Cerbino et al., 2015) (Figure 1b). No genotype/phenotype relationship was found in our AIP patients (Figure 1c).

### 3.2 Study of VP families

**FIGURE 2:**
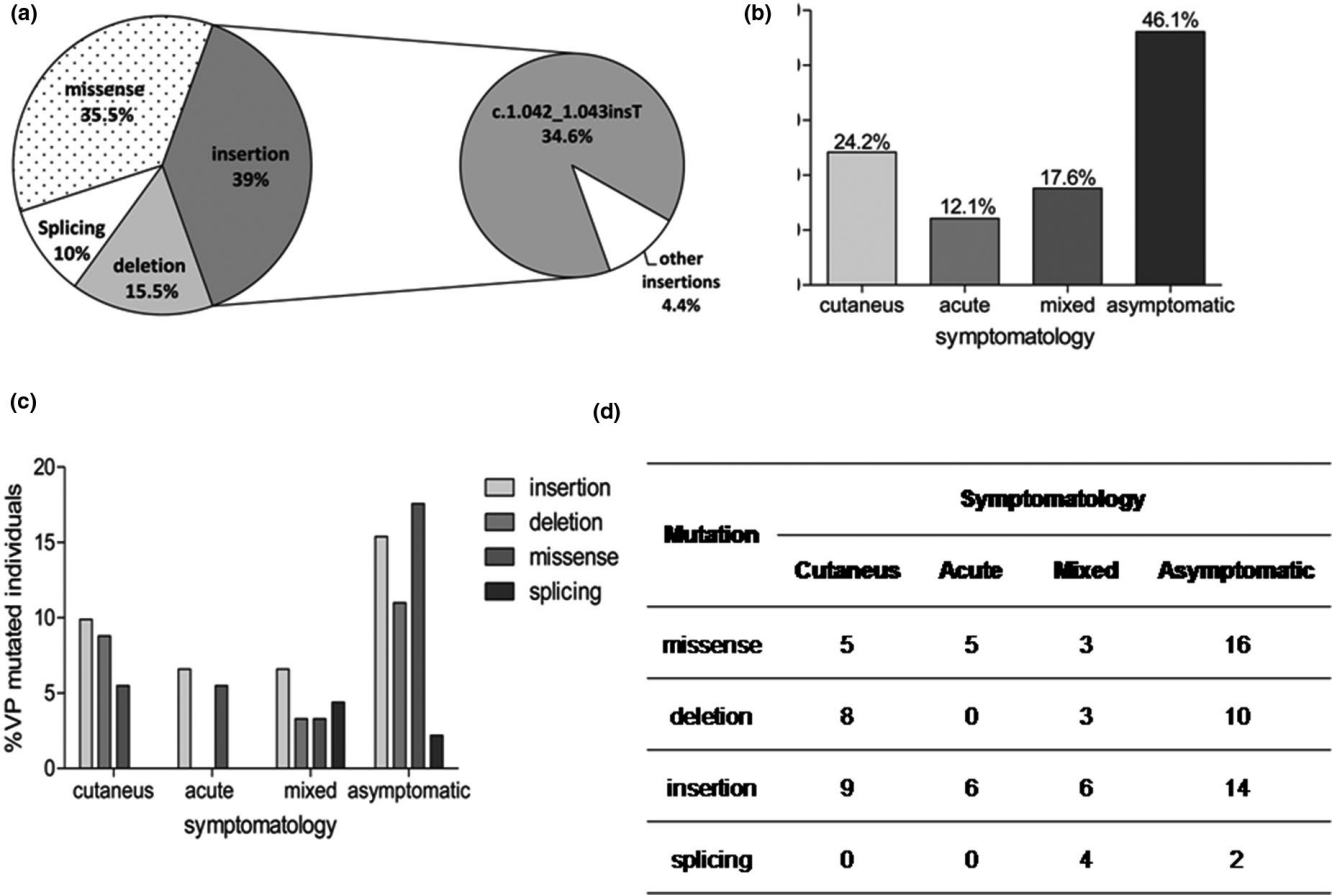
(a) Percentage distribution of the four types of VP mutations found in Argentina. The subgraph shows the distribution of c.1.042_1.043insT mutation among the total insertion mutations. (b) Percentage distribution of VP mutated individuals according to symptomatology. (c) Percentage distribution of Genotype/Phenotype correlation according to symptomatology and genetic variant. (d) Distribution of Individuals carrying VP mutation according to symptomatology and the type of genetic variant

We studied at molecular level, 36 families as VP, including a total of 132 individuals, of which, 68.94% carried the corresponding VP mutation. The results were shown in Figure 2. The insertion, c.1042_1043insT, (34.6%) was a prevalent mutation only reported in Argentina (Rossetti et al., 2008), is due to a founder effect among our population (Granata, Parera, Batlle, & Rossetti, 2015).

We did not find a genotype/phenotype correlation in VP patients (Figure 2b,c).

### 3.3 Acute porphyrias and oxidative stress

In AIP and VP patients, total oxidant capacity and oxidative damage markers as lipid peroxidation (MDA levels) and oxidative damage to proteins were assessed. We observed that there were no significant differences between patients and controls (Table 1). Regarding the antioxidant defense, levels of GSH, catalase activity and total antioxidant capacity were assessed, finding similar values between patients and controls. We determined plasma levels of total homocysteine, a marker of neurologic damage, finding no significant differences between the analyzed groups (Table 1).

**TABLE 1:** Oxidative status, antioxidant defense, and neurological damage parameters assayed

Parameter	Controls	VP	AIP
Oxidative status			
Total oxidant capacity (μmol/L)	15.19 ± 3.16	17.63 ± 4.05	19.22 ± 4.96
MDA (nmol/ml)	0.68 ± 0.05	0.75 ± 0.06	0.82 ± 0.06
Protein damage (nmol/mg)	1.81 ± 0.42	2.12 ± 0.44	2.33 ± 0.52
Antioxidant defense			
GSH (μmol/ml)	0.39 ± 0.03	0.52 ± 0.04	0.48 ± 0.04
Total antioxidant capacity (μmol/L)	939 ± 103	752 ± 124	735 ± 143
Catalase (nmol min^−1^ ml^−1^)	16.7 ± 4.7	20.5 ± 7.0	20.3 ± 7.2
Neurological damage			
Homocysteine (mmol/L)	10.7 ± 3.2	14.6 ± 4.3	15.2 ± 3.9

## 4 DISCUSSION

From the families diagnosed at the molecular level, in studied period, the percentage of latent individuals was significant and similar in both porphyrias (about 44%).

The relationship between women and men who had a clinical episode during their lifetime was 10:1, regardless of the type of AIP mutation they have. This is because women are more exposed to the factors that can trigger the typical acute attacks of AIP.

Of the amount of healthy carriers detected from the analysis of genetic studies arises the importance of doing these studies in asymptomatic individuals in diagnosed families. Thus, it is possible to advice these people about porphyrinogenic drugs or behaviors (fasting, diet, hormonal treatments, alcohol, etc.) that can lead to porphyria triggering.

In this study, it was not possible to extract data for a possible correlation between symptomatology and a type of mutation, although genotype–phenotype correlation could be established in the Finnish population (von und, 2002; zu Fraunberg, Timonen, Mustajoki, & Kauppinen,). This lack of genotype–phenotype relationship was previously reported in Argentina (Rossetti et al., 2008), as well as in other populations (Schneider‐Yin & Minder, 2006; Whatley et al., 1999). In general, we can say that apparently a given genotype is not the only determinant of the clinical presentation of porphyria (Monteiro, Abdalla, Faljoni‐Alàrio, & Bechara, 1985), in accordance with the multifactorial nature of this disease.

ALA accumulation in these porphyrias is an endogenous ROS source that can produce irreversible damage to cellular components (Monteiro et al., 1985). Furthermore, increased ROS relates to the pathogenesis of neurological dysfunction in diseases such as Alzheimer disease (AD) (Atamna & Frey, 2004). There is evidence of alterations of heme synthesis in cortical tissues from clinically and pathologically confirmed cases of AD (Dwyer, Smith, Richardson, Perry, & Zhu, 2009).

In previous reports, no alterations were detected in the antioxidant defense system in the plasma of VP patients, only a slight increase in MDA was found (Ferrer, Tauler, Sureda, Romaguera, et al., 2009). However, some alterations of oxidative stress and inflammation markers were found in erythrocytes, neutrophils, and lymphocytes of VP patients, a decrease of antioxidant capacity and increase of ROS production in neutrophils and stimulated lymphocytes (Ferrer et al., 2010; Ferrer, Tauler, Sureda, Palacín, et al., 2009; Ferrer, Tauler, Sureda, Romaguera, et al., 2009). Also, in AIP patients there are reports of high levels of plasma homocysteine, finding no relationship at all between them and the levels of heme precursors excreted in urine or clinical presentation of the disease (To‐Figueras, Lopez, Deulofeu, & Herrero, 2010).

In this work we could not find significant differences in plasma of porphyric individuals and controls in any of the parameters studied. It is likely that early diagnosis of porphyria in these patients and their constant follow‐up leads to a situation where ROS levels and the state of the antioxidant defense system are balanced. Moreover it is of note to mention that our patients, most of them without repetitive acute attacks, are successfully treated daily with a glucose infusion, vitamin B12 complex and folic acid (Wider de Xifra, Batlle, Stella, & Malamud, 1980) since we have already demonstrated that it enhanced *HMBS* activity, the deficient enzyme in AIP and also a second point of regulation in VP (De Geralnik, Rossetti, & Batlle, 1981; Ferrer, Tauler, Sureda, Palacín, et al., 2009). Although it is not known if neuronal homocysteine would be responsive to dietary measures, it was observed that an increase in dietary folate reduces plasma homocysteine. This could be explained because in brain the homocysteine could be remethylated to methionine by a 5‐methyltetrahydrofolate and vitamin B12‐dependent pathway. Moreover, by the transsulfuration pathway homocysteine is converted in cysteine for the synthesis of glutathione, involving cystathionine β‐synthase (CβS) and cystathionine γ‐lyase. Although this pathway is negligible in neural tissue because brain lacks the last enzyme, CβS is present and explains the high levels of cystathionine found in brain tissue. CβS is also a folate‐dependent enzyme and although it is activated in oxidative conditions, its redox regulation is lost in the absence of heme. The reduced CβS activity produced elevated neuronal homocysteine levels which mobilizes iron storage from ferritin, leading to a vicious circle involving disrupted iron homeostasis. This oxidative stress status is responsible for degenerative neuronal disease (Dwyer et al., 2004, 2006).

On the basis of these findings, it could be hypothesized that daily folate and vitamin B12 supplements in our patients would also decrease brain homocysteine to levels which could prevent or alleviate the oxidative damage. Furthermore, in the case of patients it is impossible to evaluate the redox status and antioxidant defenses directly into the affected organs. Assessing these parameters in biological samples such as plasma or urine, may not reflect irrefutably what happens in the organs until their deterioration being severe.

In conclusion, although we were not able to find any significant difference in the parameters measured between control group and porphyric patients, we consider that it would be relevant to carry out an extensive study to demonstrate that a profound heme deficiency in acute porphyrias could be, at least, one of the reasons accounting for the neurodegenerative damage observed in characteristic acute attacks in AHPs patients.

## CONFLICT OF INTEREST

The authors report no conflict of interest.

## AUTHOR CONTRIBUTION

Martinez M del C, Cerbino GN and Granata BX: carried out the experimental studies and analysis of data and draft the manuscript. Batlle Alcira: contributed to writing the manuscript. Parera VE: supervised biochemical studies in Argentine patients. Rossetti MV: guarantor; conception, design, and supervision of the whole work; and revising the manuscript. All authors read and approved the final manuscript.

## ETHICAL APPROVAL

Written informed consent was obtained from all patients prior to their inclusion in the study. The study protocol was approved by the Ethical Committee of the Centro de Investigaciones sobre Porfirinas y Porfirias (CIPYP—Hospital de Clínicas, CONICET‐ UBA) and was performed in accordance with the Helsinki Declaration of 1964 and its modifications (Tokyo, Japon 1975; Venece, Italy, 1983; Hong Kong, 1989; South Africa, 1996; Edimburg, Scotland, 2000; Washington, USA, 2002; Tokyo, 2004; Seul, Korea, 2008; Fortaleza, Brazil, 2013).
